# Atrial septal defect device closure and concurrent atrio‐fascicular Mahaim ablation in adult patient with Ebstein's anomaly

**DOI:** 10.1002/ccr3.6153

**Published:** 2022-07-25

**Authors:** Zahra Khajali, Atta Firouzi, Masoud Farzad Kabir, Iman Harirforoosh, Maryam Aliramezany

**Affiliations:** ^1^ Rajaie Cardiovascular, Medical, and Research Center Iran University of Medical Sciences Tehran Iran; ^2^ Tehran heart center Iran; ^3^ Cardiovascular Research Center, Institute of Basic and Clinical Physiology Sciences Kerman University of Medical Sciences Kerman Iran

**Keywords:** atrial septal defect, congenital heart disease, cribriform, mahaim accessory pathway, percutaneous

## Abstract

Ebstein's anomaly is an uncommon congenital malformation which might be associated with atrial septal defect and atrio‐fascicular Mahaim. Here, we report a known case of Ebstein's anomaly with atrial septal defect and concomitant atrio‐fascicular Mahaim pathway who underwent atrial septal defect device closure and concurrent ablation of accessory pathway.

## Graphical abstract

Careful paramedical examination is beneficial for patients by helping them to avoid undergoing an invasive treatment

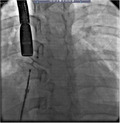

## 1 INTRODUCTION

Ebstein's anomaly is a relatively rare congenital heart disease due to tricuspid valve malformation and right ventricular myopathy.^1^ This anomaly is commonly observed with other cardiac anomalies such as atrial septal defect (ASD), ventricular septal defect (VSD), and some types of accessory pathways.^2^ Among them, the latter makes patients susceptible to developing tachyarrhythmia such as paroxysmal supraventricular tachycardias, atrial fibrillation, and atrial flutter which have been reported in 25 to 30% of the patients.^3^

Atrio‐fascicular Mahaim (AFM) pathway is one of possible associated conditions diagnosed in Ebstein's anomaly. AFM is described as the presence of conducting pathways between AV node in RV and right bundle branch.^4^ The association between Ebstein's anomaly and AFM is due to Mahaim fibers' proximal end being located near the lateral tricuspid annulus.^5^ Due to the risk of arrhythmia, electrophysiological study is recommended before therapeutic interventions for Ebstein's anomaly.

ASD is another associated lesion with Ebstein's anomaly which needs attention. In these patients, the shunt is usually right to left, which increases with the age of the patient; the severity of the tricuspid regurgitation and right ventricular function. Closure of this defect can lead to increased central venous pressure and decreased cardiac output due to reduced left ventricular preload.^6^ The age of onset of symptoms is usually different and depends on the severity of the tricuspid regurgitation; right ventricle function and associated abnormalities. Hence, careful hemodynamic examination is recommended before deciding to correct associated anomalies in these patients because, the appropriate time for proper intervention and treatment is a challenging issue and is related to many factors.^2^

In this paper, we report the case of a 32‐year‐old woman, a known case of Ebstein's anomaly with large ASD and concomitant atrio‐fascicular Mahaim pathway who successfully underwent ASD device closure with Cribriform Amplatzer device and ablation of accessory pathway.

## 2 CASE PRESENTATION

A 32‐year‐old female, known case of Ebstein's anomaly and concurrent Atrial Septal Defect (ASD), referred to our center with complaint of frequent palpitation attacks. The patient was under medical treatment and regular follow‐up due to her stable condition. In basic electrocardiography, we observed normal sinus rhythm and no evidence of abnormalities.

**FIGURE 1:**
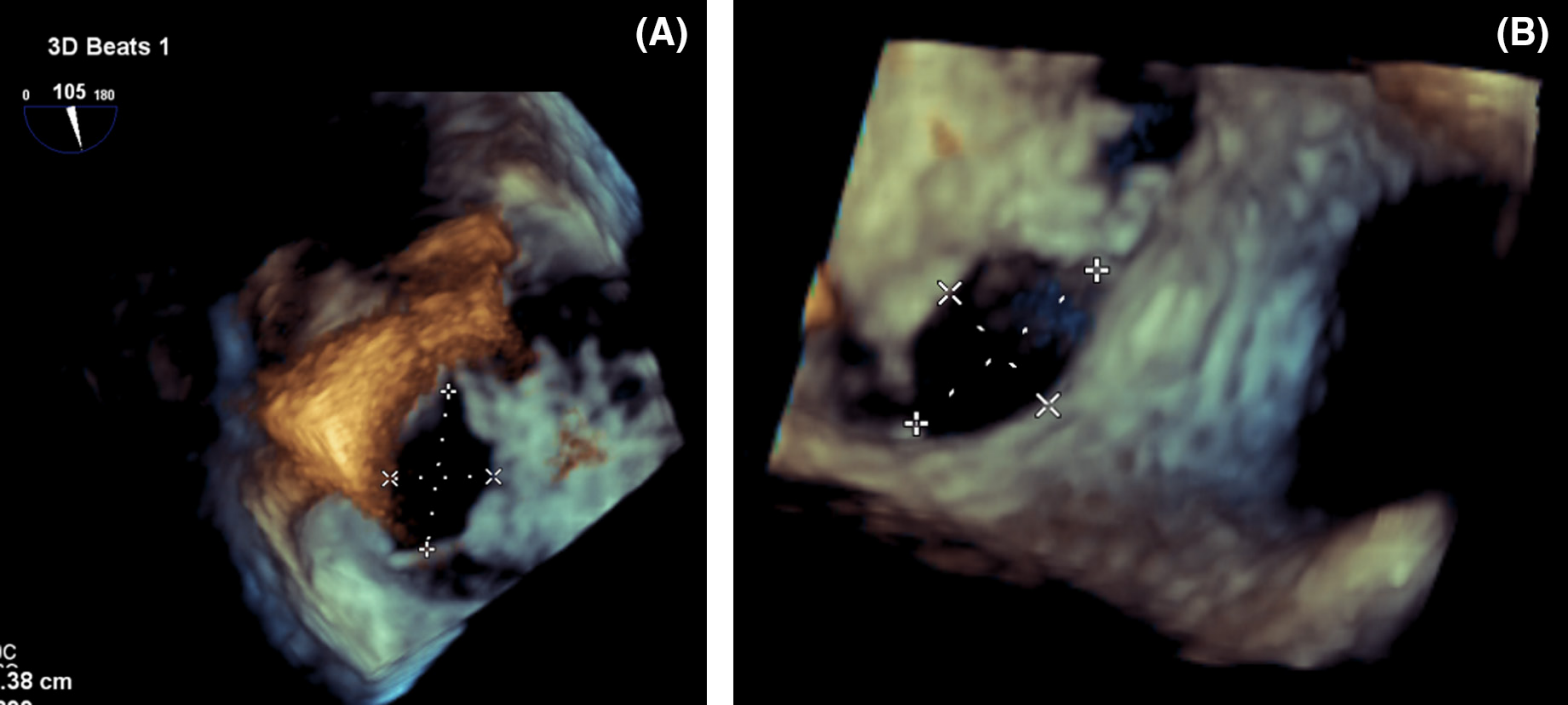
A,B: multiple ASD in 3D echocardiography

For better evaluation, three‐dimensional (3D) trans‐esophageal echocardiography (TEE) was done and revealed four secundum type ASDs in different levels of inter‐atrial septum (IAS) (1.4 cm, 0.6 cm, 0.4 cm, 0.7 cm), with multiple small fenestrations that caused left to right shunt with appropriate rims for device closure (postero‐superior rim:0.6 cm, postero‐inferior rim: 0.4 cm, antero‐inferior rim: 2.3 cm) and also there was a large stretched patent foramen ovale (PFO) (0.3 cm*1.23 cm) with distance of about 1.1 cm from ASD.(Figure 1). Other findings of echocardiography were as follows: normal left ventricle (LV) size with mild systolic dysfunction; severe right ventricle (RV) enlargement and systolic dysfunction; functional RV/anatomical RV ratio:70%, fractional area change (FAC):30%; apical displacement of tricuspid valve (TV) septal leaflet: 11 cm/m2 and posterior leaflet: 3.3 cm/m2 with elongation of anterior leaflet. Mild to moderate tricuspid regurgitation (TR) without pulmonary hypertension.

Due to history of frequent palpitation, after consultation with electrophysiology expert team, she was candidated for electrophysiological study (EPS). In EPS, changes of electrocardiography (ECG) to left bundle branch bloke (LBBB) pattern during rapid atrial pacing with negative HV and the most fused AV and Mahaim potential in the postero‐lateral part of TV annulus area were observed. Furthermore, two different narrow QRS tachycardia were also induced; the first one was the atrial tachycardia (AT) from right postero‐septal area with cycle length of about 285 ms, HV and AH of about 45 and 115 ms, respectively, with retrograde atrial activation with earliest A in the HBE. The second was AT from infra‐crista region with cycle length of about 330 ms and HV and AH of about 45 and 115 ms, respectively, with retrograde atrial activation and with earliest A in high right atrium (HRA). Finally, EPS revealed that she had Mahaim accessory pathway (Atrio‐fascicular type) and Atrial tachycardia from postero‐septal and infra‐crista regions; all of them were successfully ablated. Moreover, because of atrial fibrillation (AF) rhythm, she received external electrical DC shock which fully converted AF rhythm to sinus rhythm.

**FIGURE 2:**
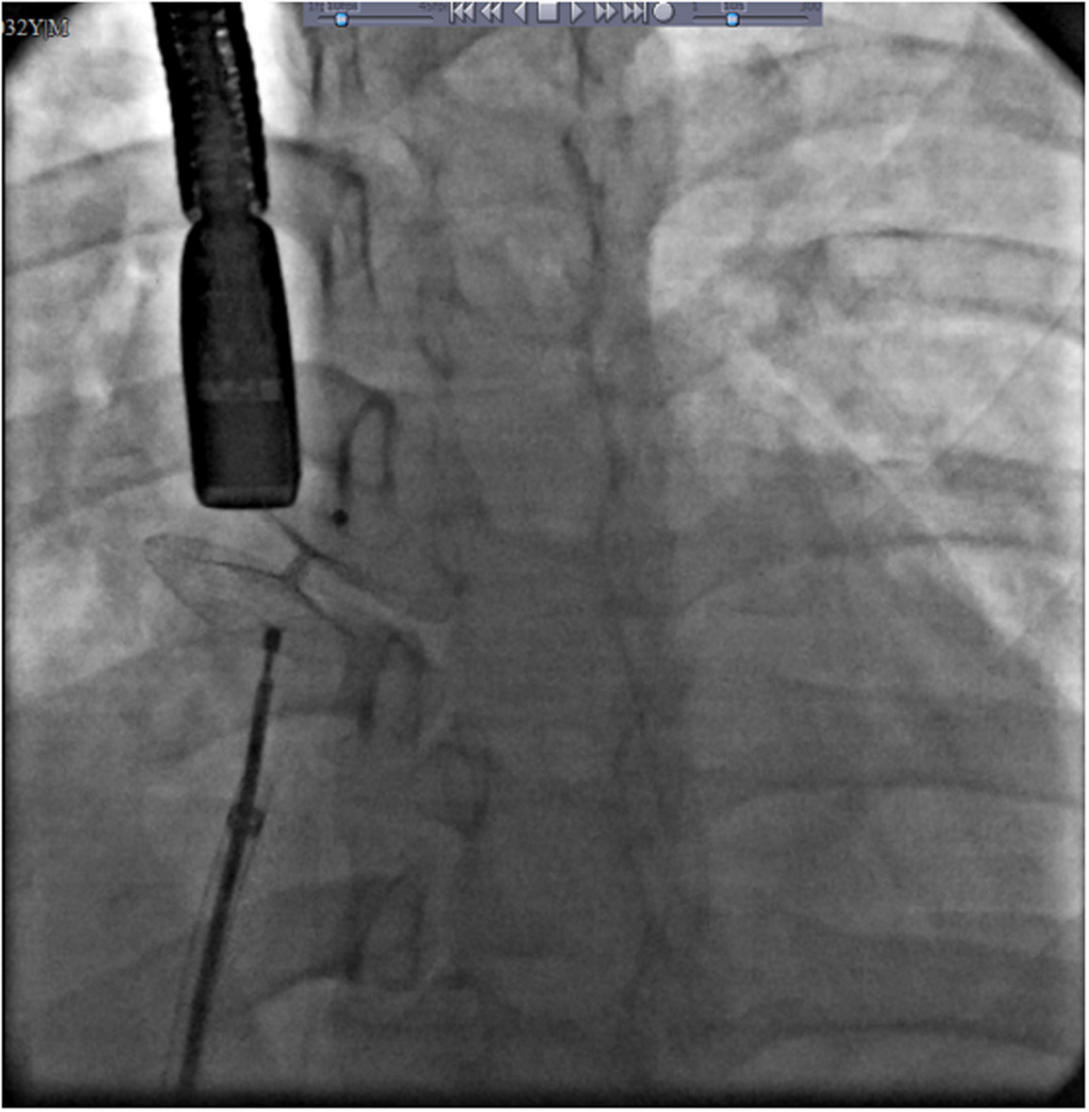
proper position of Cribriform device in angiographic view

After successful ablation of accessory pathways, since morphological properties of her ASD made her suitable for device closure, she underwent diagnostic and therapeutic catheterization. During catheterization, hemodynamic study showed evidence of left to right shunt via ASD with ratio of pulmonary to systemic blood flow (QP/QS): 1.6. Patient hemodynamic and oximetry data is shown in Table 1. Also, left ventricular (LV) injection showed normal LV size with mild systolic dysfunction; abnormal septal motion and also abnormal shape of LV in favor of Ebstein's anomaly. ASD closure was performed by an Amplatzer Multi Septal Occluder Cribriform device of 35 mm without any complication. There remained only a small posterio‐superiorly placed ASD about 2 mm which had no hemodynamically significant residual shunt. Figure 2 shows the final successful closure of multiple ASDs using a single Cribriform Amplatzer device.

**TABLE 1:** Patient hemodynamic and oximetry information

Pressure (mmHG)	Saturation(percent)
LV:120/0–12	AO:95
RV:30/0–8	SVC:70
RA:8	MRA:80
PA:25/15	IVC:75
	PA:81

## 3 DISCUSSION

Ebstein's anomaly is an uncommon congenital malformation with different presentations^1^ due to its heterogenic nature. These patients can remain asymptomatic for years, but the common symptoms are abnormal heart rhythms; shortness of breath; fatigue and bluish discoloration of the skin.^7^ A significant issue in patients with Ebstein's anomaly is determining the appropriate time for therapeutic intervention. Asymptomatic patients only need regular follow‐ups and evaluation for the onset of symptoms. One of the most common manifestations of the disease in adulthood is arrhythmia due to accessory pathways. Atrial fibrillation, atrial flutter, and ectopic atrial tachycardia are usually seen in 40% of newly diagnosed adult patients. A distinct subgroup of these atrioventricular accessory pathways is called Mahaim fibers which is observed in less than 2% of the normal population and up to 13% of the patients with Ebstein's anomaly.^1^

In our case, due to the history of palpitations and the possibility of preexcitation syndrome, at first, electrophysiological study was performed which demonstrated atrio‐fascicular Mahaim pathway in addition to atrial fibrillation and atrial tachycardia which is a relatively rare association in Ebstein's anomaly. Furthermore, in these patients, antiarrhythmic medication not only fails to effectively prevent tachycardia recurrences, but also carries a high potential for aggravating preexisting disturbances of physiological impulse generation or conduction and does not seem to be attractive to a mainly young population of patients requiring long‐term treatment. Hence, catheter ablation of cardiac arrhythmias particularly those related to presence of accessory pathways is the best choice for management of these patients.^3^

Another cardiac anomaly known to be associated with Ebstein's anomaly is ASD with the prevalence of 80%–94%.^8^ Although in most patients there is a right‐to‐left shunt via ASD,^6^ but our case according to different paraclinical studies, had significant left to right shunt that need intervention and with regard to lack of other cardiac anomalies, we candidated her for trans‐catheter closure. However, if other heart defects are acknowledged, the only approach is surgery.^9^

Today, trans‐catheter approach is the gold standard method for ASD closure in non‐Ebstein patients. For Ebstein patients who have traditionally been managed by surgical approaches, trans‐catheter closure of ASDs seems more attractive due to fewer associated complications including arrhythmias resulted from surgical scars.^10^

Based on 3D echocardiography finding, she had multiple fenestrated ASDs, and for closure of such ASDs, different strategies are available. Among them, we used Amplatzer Cribriform Multi‐Fenestrated Septal Occluder (Cribriform ASO) (Abbott Structural), which is a non‐self‐centering device that have two large equal sized discs with small linking waist diameter that can cover more than one adjacent defect.^11^ In this way, we usually can close all defects with a single device and often in some cases, a small residual defect is left behind with hemodynamically nonsignificant shunt.^12^

Another way for closure of fenestrated secundum ASD that accounts for 10% of all ASDs is simultaneous implantation of two or more devices or use of atrial septostomy with balloon and tear the strands and then placing a single larger atrial septal Occluder (ASO). Although these two methods have been successfully performed in some cases, they have side effects such as prolonged procedure time, risk of erosion and embolization, which are not seen when using Cribriform device.^11^

In general, we noticed concurrent presentation of Ebstein's anomaly with multiple small ASDs and a less common accessory pathway known as Mahaim accessory pathway. Beside these, we also challenged with a different kind of tachyarrhythmia known as Atrial tachycardia (AT).

Our decision to perform this procedure was based on the patient's symptoms and the findings of echocardiography (appropriate rims and defect size) and angiography (significant left‐to‐right shunt) and in our patient, only a small postero‐superiorly ASD of about 2 mm remained, which had no hemodynamically significant residual shunt and we preferred to keep this defect as a backup because of the underlying patient's anatomy.

This was the first time we tried to correct multiple ASDs with such an anatomy in a known case of Ebstein's anomaly by a cribriform device. There are few reports in the literature of trans catheter closure of the septum defect in Ebstein patients, which includes a small ASD with left to right shunt PFO.^10^

## 4 CONCLUSION

Our experience with the reported case indicated that percutaneous device closure of multiple ASDs after percutaneous electrophysiological study is useful in management of patients even with complex anatomy and conductive pathway disorders and this should be as an available and minimally invasive approach on the table for management of these patients. It is worth mentioning that the important issue in these patients is the accurate anatomical and hemodynamic evaluation with different paraclinical methods. In general, timely decision and appropriate patient selection and type of intervention can help to reduce complications and improve the prognosis of patients.

## AUTHOR CONTRIBUTIONS

MA, ZK, and AF managed the patient. MA drafted the paper. IH and FM revised the draft. All authors read and approved final version of the paper.

## ETHICAL APPROVAL

We took informed consent of the patient for reporting this case, additionally we ensured that her information would not be disclosed.

## CONSENT

Written informed consent was obtained from the patient to publish this report in accordance with the journal's patient consent policy

## Data Availability

Data sharing is not applicable to this article as no new data were created or analyzed in this study.
